# SPIRE: a Searchable, Planetary-scale mIcrobiome REsource

**DOI:** 10.1093/nar/gkad943

**Published:** 2023-10-28

**Authors:** Thomas S B Schmidt, Anthony Fullam, Pamela Ferretti, Askarbek Orakov, Oleksandr M Maistrenko, Hans-Joachim Ruscheweyh, Ivica Letunic, Yiqian Duan, Thea Van Rossum, Shinichi Sunagawa, Daniel R Mende, Robert D Finn, Michael Kuhn, Luis Pedro Coelho, Peer Bork

**Affiliations:** Structural and Computational Biology Unit, European Molecular Biology Laboratory, 69117 Heidelberg, Germany; Structural and Computational Biology Unit, European Molecular Biology Laboratory, 69117 Heidelberg, Germany; Structural and Computational Biology Unit, European Molecular Biology Laboratory, 69117 Heidelberg, Germany; Structural and Computational Biology Unit, European Molecular Biology Laboratory, 69117 Heidelberg, Germany; Structural and Computational Biology Unit, European Molecular Biology Laboratory, 69117 Heidelberg, Germany; Institute of Microbiology, Department of Biology and Swiss Institute of Bioinformatics, ETH Zurich, Vladimir-Prelog-Weg 4, 8093 Zurich, Switzerland; Biobyte solutions GmbH, Bothestr. 142, 69117 Heidelberg, Germany; Institute of Science and Technology for Brain-Inspired Intelligence, Fudan University, Shanghai 200433, China; Structural and Computational Biology Unit, European Molecular Biology Laboratory, 69117 Heidelberg, Germany; Institute of Microbiology, Department of Biology and Swiss Institute of Bioinformatics, ETH Zurich, Vladimir-Prelog-Weg 4, 8093 Zurich, Switzerland; Department of Medical Microbiology, Amsterdam University Medical Centers, Amsterdam, The Netherlands; European Bioinformatics Institute (EMBL-EBI), European Molecular Biology Laboratory, Wellcome Genome Campus, Hinxton, United Kingdom; Structural and Computational Biology Unit, European Molecular Biology Laboratory, 69117 Heidelberg, Germany; Institute of Science and Technology for Brain-Inspired Intelligence, Fudan University, Shanghai 200433, China; Centre for Microbiome Research, School of Biomedical Sciences, Queensland University of Technology, Translational Research Institute, Woolloongabba, Queensland, Australia; Structural and Computational Biology Unit, European Molecular Biology Laboratory, 69117 Heidelberg, Germany; Department of Bioinformatics, Biozentrum, University of Würzburg, 97074 Würzburg, Germany; Max Delbrück Centre for Molecular Medicine, 13125 Berlin, Germany

## Abstract

Meta’omic data on microbial diversity and function accrue exponentially in public repositories, but derived information is often siloed according to data type, study or sampled microbial environment. Here we present SPIRE, a Searchable Planetary-scale mIcrobiome REsource that integrates various consistently processed metagenome-derived microbial data modalities across habitats, geography and phylogeny. SPIRE encompasses 99 146 metagenomic samples from 739 studies covering a wide array of microbial environments and augmented with manually-curated contextual data. Across a total metagenomic assembly of 16 Tbp, SPIRE comprises 35 billion predicted protein sequences and 1.16 million newly constructed metagenome-assembled genomes (MAGs) of medium or high quality. Beyond mapping to the high-quality genome reference provided by proGenomes3 (http://progenomes.embl.de), these novel MAGs form 92 134 novel species-level clusters, the majority of which are unclassified at species level using current tools. SPIRE enables taxonomic profiling of these species clusters via an updated, custom mOTUs database (https://motu-tool.org/) and includes several layers of functional annotation, as well as crosslinks to several (micro-)biological databases. The resource is accessible, searchable and browsable via http://spire.embl.de.

## Graphical Abstract

**Graphical Abstract:**
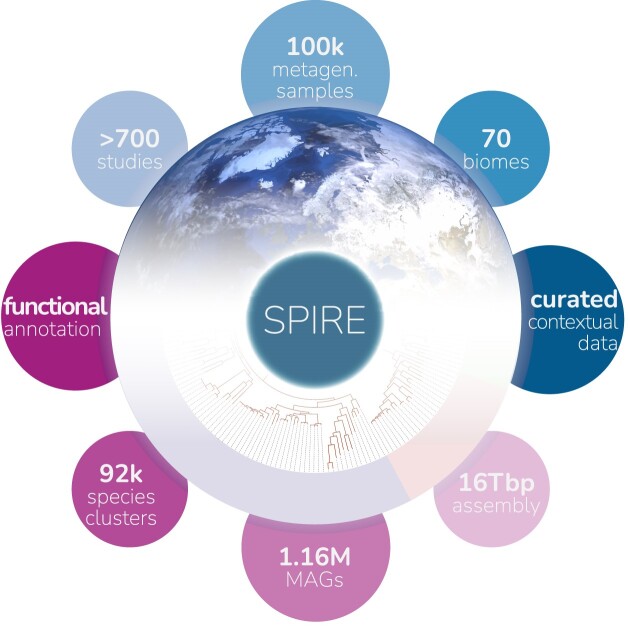

## Introduction

Life on Earth is dominated by microbes: bacteria, archaea and small eukaryotes shape our world by driving biogeochemical cycles across ecosystems (1), they enable macroscopic life as plant and animal symbionts (2), and they represent by far the greatest biodiversity among known life (3). Yet most of this diversity remains biological ‘dark matter’ (4): although meta’omic techniques enable their study directly from sequencing data, the vast majority of microbes eludes laboratory cultivation and only a small fraction of the functional space encoded by microbial genes has been characterized (5,6). While sampling efforts have increased exponentially and generated petabytes of data in recent years (7), most major microbial habitats remain understudied to the extent that almost every newly sequenced metagenome adds ‘novel’ species (as inferred from metagenome-assembled genomes, MAGs) and thousands of ‘novel’ genes of unknown function to the census (8).

The bulk of metagenomic data is generated in individual studies to address specific research questions. Heterogeneity in sample preparation (9), sequencing protocols and bioinformatic processing workflows (10,11) complicate comparisons of findings across studies. Several initiatives have sought to integrate and consolidate datasets by re-processing them using consistent pipelines. For example, QIITA (12), MGnify (7) or the Microbe Atlas Project (https://microbeatlas.org/) host millions of amplicon samples, whereas other projects, such as curatedMetagenomicData (13), GMrepo (14) and the OceanMicrobiomicsDatabase (15), focus on taxonomic and functional profiles of human-associated or ocean metagenomes. Large MAG catalogs for multiple biomes are hosted online as part of the DOE’s IMG/M (16) and EBI’s MGnify (7) resources. Moreover, the Genome Taxonomy Database (GTDB, 17) has advanced the field by consistently organizing both isolate genomes and quality-filtered MAGs into a common prokaryotic reference tree that guides standardized, phylogeny-informed taxonomies (18–20). The GTDB encompasses 85 205 species-level genome clusters across 181 phyla (as of release r214, April 2023), two thirds of which are represented only by MAGs, while also providing widely used tools for genome quality control (21) and taxonomic classification (22). Overall, existing resources focus on either providing large gene or genome catalogs, on functional and taxonomic profiling, or on harmonizing contextual data given heterogeneous data submission and annotation practices, and are often restricted to individual microbial habitats or cordon data on different habitats off into distinct subsets.

Here we introduce SPIRE, a Searchable, Planetary-scale, Integrated mIcrobiome REsource to study microbial diversity and function at global habitat, geographical and phylogenetic scales. As detailed below, SPIRE version1 encompasses 99 146 consistently processed whole-genome shotgun metagenomic samples from 739 distinct studies, integrated across environments and amended with manually curated contextual data, based on a newly developed lightweight ‘microntology’ of 92 terms describing microbial habitats and lifestyles. SPIRE combines 1.16 million newly constructed MAGs of medium or high quality (23) with the 907k high-quality reference genomes in proGenomes3 (24), clustered into 133 402 species-level genome clusters, 78 804 of which are unclassifiable at species level using current tools (22). Species clusters are profilable using mOTUs (25) via an updated custom database and pre-computed taxonomic profiles across all 99k metagenomic samples will be released as part of the resource. SPIRE further comprises 35 billion metagenomically called open reading frames (ORFs) with various layers of functional annotation, linked to clusters in the Global Microbial Gene Catalogue (GMGC, 8). SPIRE provides consistent integration of these heterogeneous data modalities and is designed to interoperate with other (micro-)biological resources, such as proGenomes (24, https://progenomes.embl.de), the GMGC (8, https://gmgc.embl.de), eggNOG (26, http://eggnog6.embl.de) and metaMap (https://metamap.biobyte.de/), among others. The resource can be accessed, browsed, and searched via https://spire.embl.de.

## Database construction and characteristics

### Metagenome collection and dataset curation

The core dataset underlying SPIRE was defined using a semi-automatic process, combining three data sources: (i) samples in the European Nucleotide Archive (ENA) meeting the criteria ‘library_source = METAGENOMIC AND library_strategy = WGS AND instrument_platform = ILLUMINA AND base_count >= 10^9 AND average read length >= 100’ were selected from all projects where >=20 samples satisfied the above criteria as of Sep 30th 2022; (ii) metagenomic samples available via the JGI’s IMG/M resource (27) on Sep 30th 2019 (to comply with JGI data policies and embargo periods); (iii) manually selected ‘allowlisted’ studies of particular interest (e.g. providing data on exotic environments). For the resulting list, ENA project accessions were manually matched to publications where possible; in case of data submitted by the JGI, where each metagenomic sample is associated with a distinct project accession, ‘studies’ were defined based on matched publications and as consistent groups based on sample metadata provided via IMG/M.

The metagenomic sample set was further filtered and curated by (i) removing amplicon and isolate genome sequencing datasets erroneously annotated as shotgun metagenomes; (ii) identifying and removing erroneously submitted datasets (e.g. where both mates in ‘paired end’ data were identical); (iii) identifying and removing duplicates (submitted under distinct project or sample accessions); (iv) removing samples from controlled experimental setups (e.g. laboratory mice, pathogen challenges or defined *in vitro* communities); (v) flagging special cases such as microcosms, paleobiological samples or pre-enriched samples; (vi) resolving misfits with the European Nucleotide Archive (ENA) and Sequence Read Archive (SRA) data model, e.g. if distinct biological samples were erroneously submitted under the same biosample accession, but distinct experiment or run accessions; (vii) identifying and combining technical replicates (distinct experiment accessions) for the same biological sample. For the resulting list, raw sequencing data was downloaded from the ENA.

Following these steps, the final dataset in SPIRE comprises 99 146 metagenomic samples across 739 distinct studies.

### Curation of contextual data and overview of sampled environments

**Figure 1.**
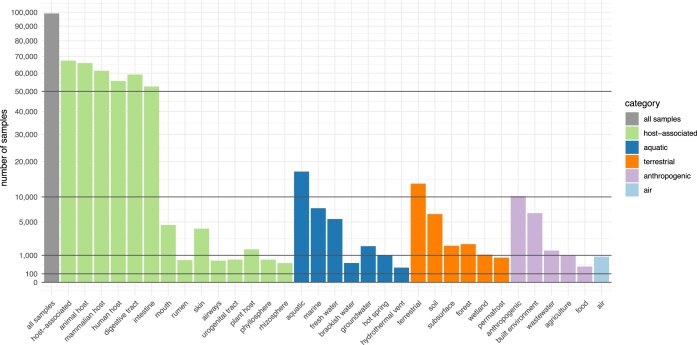
Overview of sampled habitats in SPIRE, as a subset of annotated ‘microntology’ terms (see table S1). Microntology terms are assigned using a ‘multi-tag’ system, meaning that individual samples can be annotated with multiple terms of varying granularity and redundantly within a flat hierarchy (e.g. a human fecal metagenome will be annotated as ‘host-associated, animal host, mammalian host, human host, digestive tract, intestine’, whereas a mangrove-associated sample carries tags from both the ‘aquatic’ and ‘terrestrial’ term space, while moreover possibly being annotated as ‘host-associated, plant host’). Shown above is the total number of samples annotated to a subset of microntology terms under this system.

Contextual data for each metagenomic sample was sourced (i) via annotation fields in ENA, (ii) via IMG/M metadata tables where applicable and (iii) directly from matched publications. Information was consolidated into common fields (e.g. latitude and longitude data were manually harmonized across different submitted formats). All samples were manually annotated against a newly developed ‘*microntology*’ (see Table S1), a shallow and lightweight ontology of 92 terms to describe microbial habitats and lifestyles, crosslinked to terms in established resources such as the EnvO (28) or UBERON (29) ontologies. SPIRE sample annotation uses a ‘multiple tag’ system, meaning that each sample is described using a combination of concurrent tags, rather than one specific term in a (deep) hierarchy, allowing an annotation with increased flexibility, yet compatibility to established ontologies. As a result, for example, 68% of the ∼100k samples in SPIRE are annotated as ‘host-associated’ (66.5% as animal-associated, 56% as human-associated, 1.5% as plant-associated); 17% are aquatic samples (including 7.6% marine and 5.5% fresh water); 13.5% are terrestrial (including 6.4% soil samples); 10.3% are from anthropogenic or human-impacted environments (including 6.6% from built environments); see Figure 1 for details. Moreover, data included in SPIRE cover pole-to-pole latitudes, with samples from ∼200 countries and territories.

### Metagenomic sequence processing

Data processing was implemented in a Nextflow pipeline (30) to enable robustness and reproducibility. Downloaded sequence data was quality-trimmed and filtered using NGless (31) as described previously (8). Reads were assembled into contigs using megahit v1.2.9 (32) with default parameters, separately for each sample, resulting in a total of 210M contigs and a total assembly length of 16 Tbp. From these, a total of 35 billion open reading frames (ORFs) were called using prodigal v2.6.3 (using -p meta) (33) and further processed as described below. Sequences of tRNA genes were called using tRNAscan v2.0 (34) using the ‘general’ model; rRNA genes were called using Barrnap v0.9 (https://github.com/tseemann/barrnap); putative CRISPR sequences were identified using MinCED (https://github.com/ctSkennerton/minced).

### Metagenome-assembled genomes (MAGs)

MAGs were binned from pre-filtered contig sets per sample (length >= 1000 bp) using metaBAT2 (v2.12.1) (35), resulting in a total of 3 023 270 genome bins which were further filtered to 1.16 million bins of ‘medium or high quality’ (CheckM2-inferred completeness >= 50% and contamination <= 10% (21); passing default clade consistency score filters and <= 5% estimated contamination in GUNC (36); CheckM2 v0.1.3, GUNC v1.0.1). Of these, 73.3% were mapped to the 41 171 species-level clusters of high quality reference genomes in proGenomes3 (24) based on a two-step procedure: (i) extraction of 40 ‘specI’ marker genes using fetchMG (37), followed by MAPseq-mapping (38) of each marker gene with parameters calibrated for high specificity and a consensus call across hits per query MAG; (ii) fastANI-derived (39) Average Nucleotide Identity (ANI) of >= 95% to species representative genomes. The remaining 309 020 unmapped MAGs were clustered into 92 134 species-level groups in a two-step procedure: (i) single linkage preclustering at 90% whole genome ANI using mash (40); (ii) resolution of mash pre-clusters into 95% ANI average linkage clusters using fastANI (39) and fastcluster (41). A full list of species clusters included in SPIRE with consensus taxonomic classifications is included as Supplementary Table S2. In an independent approach, all 1.16 million medium and high quality bins were mapped against the mOTUs 3.1 database (25) and unmapped MAGs were clustered into 84 287 novel mOTUs based on 10 marker genes, resulting in a novel, extended mOTU-profilable database. The ANI- and mOTU marker gene-based partitions of the data were highly concordant at an Adjusted Mutual Information (42,43) of 0.98.

**Figure 2.**
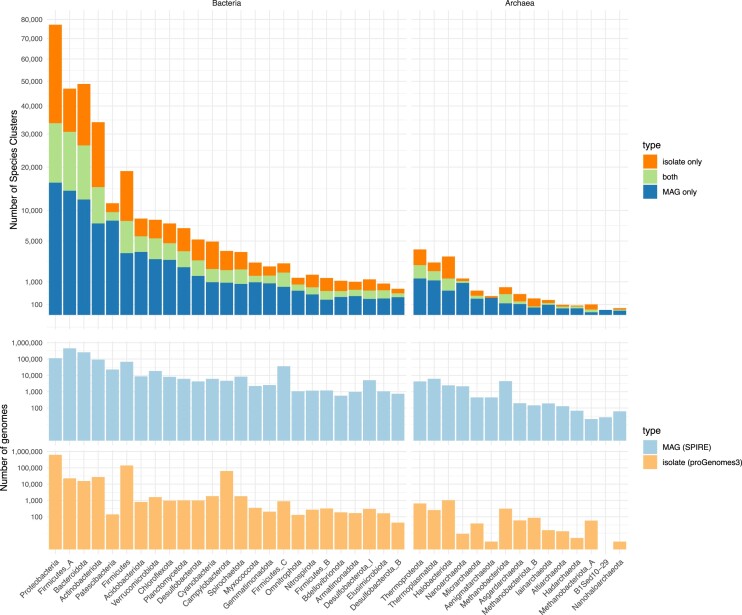
Representation of taxonomic groups covered in SPIRE. Shown are the total number of species clusters (top) and total number of genomes (bottom) for the largest 25 bacterial and largest 15 archaeal phyla represented in SPIRE. Orange hues indicate clusters and genomes of isolates, as downloaded from proGenomes3 (progenomes.embl.de; ‘isolate only’). Blue hues indicate clusters and genomes introduced in SPIRE (‘MAGs only’). Green indicates species clusters that contain both isolate genomes and MAGs. See Supplementary Table S2 for taxonomic classifications of all species clusters included in SPIRE.

All SPIRE MAGs were taxonomically classified using gtdb-tk v2.11 against release r207 (22) and consensus taxonomy for species clusters at each taxonomic level was assigned based on a majority vote, with manual resolution of a few remaining conflicting labels. The 92 134 MAG-based species clusters were classified into 178 different phyla (926 clusters representing 1 185 MAGs remained unclassified at phylum level) and 11 082 named species (79 782 clusters representing 198 384 MAGs remained unclassified at species level; Figure 2). This large proportion of ‘novel’ species relative to the GTDB may in part be due to a conservative parametrization of the gtdb-tk classifier (favoring specificity over sensitivity), but it indicates that SPIRE covers a vast diversity of previously uncharacterized and undescribed microbial diversity. Notably, 28 856 SPIRE clusters unclassified at species level contain more than a single genome.

### Functional annotation

Detection of orthologs and inference of putative function for metagenomically-called ORFs (see above) were performed using eggNOG-mapper v2 (44,45). ORFs were further annotated for putative roles in antibiotics resistance using DeepARG (46) and abricate v1.0.1 (https://github.com/tseemann/abricate) against the MEGARes (version 2020-04-19, 47) and VFDB (version 2020–04-19, 48) reference databases.

### Database design

SPIRE relies on a mongoDB database as its foundation. Within this system, a repository of samples/MAGs and their attributes is stored. This data can be conveniently accessed through the web-based interface. Structured data such as annotation of genes and genomes is stored in a relational database management system to allow complex and time efficient queries.

### Website

SPIRE is accessible, browsable, searchable and downloadable via spire.embl.de. The main access modes are *by habitat/sample* (searching based on accessions or metadata tags), *by taxon* (based on clade names and species-level clusters) and *by genome* (individual genomes within clusters). These modes are inter-accessible (e.g. browsing from a sample to a specific taxon observed therein, for which then multiple genomes can be accessed) and at each level, link-outs to relevant independent or third party databases are provided. We invite user contributions, suggestions for improvements and bug reports under spire.embl.de/contribute.

### Outlook

Given the exponential growth of publicly available metagenomic data, we anticipate biennial updates of the underlying data for SPIRE. We will continue to develop and update the processing pipeline to address rising computational demands and integrate novel or improved tools. Moreover, we will seek to extend the range of available functional annotations at gene and genome level, within the limits of computational scalability. Finally, and most importantly, we will continue to further integrate SPIRE with other resources such as proGenomes (24), eggNOG (26), the GMGC (8) and other ongoing efforts.

## Discussion

SPIRE provides the largest sets of consistently processed metagenomes, newly generated MAGs and profilable microbial species clusters to date. Combined with a high degree of curation and integration of various data modalities (MAGs, contigs, genes, profiles, etc.), SPIRE is the most comprehensive resource available to study microbial diversity and function. Covering a broad range of habitats and geography, SPIRE enables true ‘planetary-scale’ analyses of microbiomes across various environments, including so far understudied ones. At the same time, SPIRE encompasses large amounts of ‘novel’, previously undescribed microbial diversity both at the gene and genome level. We are confident that SPIRE will enable and simplify a wide range of analyses for end users, ranging from the characterization of individual taxa or gene clusters of interest against a global data canvas, to truly ‘planetary-scale’ studies of microbial life across habitats and phylogeny.

## Supplementary Material

gkad943_supplemental_files

## Data Availability

All raw data underlying SPIRE v1 is publicly available via the European Nucleotide Archive and/or the Sequence Read Archive. No new sequencing data was generated for this study. The derived and curated data described above is freely accessible and downloadable via spire.embl.de. SPIRE is released under a Creative Commons Attribution-ShareAlike 4.0 International License.

## Notes

Present address: Pamela Ferretti, Section of Genetic Medicine, Division of Biological Sciences, University of Chicago, Chicago, USA.

Present address: Oleksandr M. Maistrenko, Royal Netherlands Institute for Sea Research (NIOZ), Department of Marine Microbiology & Biogeochemistry, 1797 SZ, ’t Horntje (Texel), The Netherlands.
