# Supplementary material for: SPIRE: a Searchable, Planetary-scale mIcrobiome REsource
Source: Nucleic Acids Res. 2023 Oct 28;52(D1):D777–83. doi: 10.1093/nar/gkad943 (PMC10767986; doi:10.1093/nar/gkad943)
Supplement: gkad943_supplemental_files [file gkad943_supplemental_files.zip › SPIRE S table legends.docx]

**Supplementary Table Legends**

**S1: Microntology overview.** A list of terms included in the initial version (v01) of the newly developed *microntology*. Terms are organised by ‘category’, i.e. the highest level of biome (e.g., aquatic, terrestrial, host-associated, etc.), physicochemical (e.g., temperature, ph, oxygen level) or human host (age group, birth term) descriptors for microbial samples. Within these categories, terms are organised into a flat hierarchy. Cross-links to established ontologies (EnvO, UBERON) and host NCBI taxonomy IDs are provided, as well as additional descriptive comments to define individual terms. Microntology annotations in SPIRE follow a ‘multiple tag’ logic where a given sample is annotated with a combination of terms, rather than choosing a single most descriptive term. In consequence, some microntology terms are inherently cross-linked: e.g., a mangrove sample (microntology term ‘terrestrial:wetland:mangrove’) will automatically receive the further tags ‘aquatic:lentic’ and ‘aquatic:littoral’. These cross-links are provided in the column ‘pull_term’.

**S2: SPIRE species-level cluster metadata.** A list of all species-level genome clusters included in SPIRE, spanning both specI clusters as defined in proGenomes3 (progenomes.embl.de) to which SPIRE MAGs were mapped (‘specI_v4_XXXXX’), and newly generated de novo clusters at 95% average nucleotide identity (see Methods; ‘spire_v1_095_XXXXXXXXX’). For each cluster, the number of medium or high quality MAGs in SPIRE (‘size.spire’), the number of reference genomes in proGenomes3 (‘size.pg3’), total number of genomes (‘size.combined’), and consensus taxonomic classification at domain, phylum, class, order, family, genus and species level are provided.
